# Effects of High Doses of Cholecalciferol in Normal Subjects: A Randomized Double-Blinded, Placebo-Controlled Trial

**DOI:** 10.1371/journal.pone.0102965

**Published:** 2014-08-28

**Authors:** Birgitte Nygaard, Niels Erik Frandsen, Lisbet Brandi, Knud Rasmussen, Ove Vyff Oestergaard, Lars Oedum, Hans Christian Hoeck, Ditte Hansen

**Affiliations:** 1 Department of Medicine, Roskilde University Hospital, Roskilde, Denmark; 2 Department of Clinical Biochemistry, Roskilde University Hospital, Roskilde, Denmark; 3 Center for Clinical and Basic Research, Aalborg, Denmark; Universidade de São Paulo, Brazil

## Abstract

**Background:**

Vitamin D repletion with high doses of vitamin D is often recommended to patients and healthy subjects. The safety, especially concerning changes in urinary calcium excretion is of great importance.

**Methods:**

In a double-blinded, placebo-controlled study in 40 healthy volunteers, we examined the changes in mineral metabolism during supplementation with 3000 IU of oral cholecalciferol daily during 4 months.

**Results:**

Both 25(OH)vitamin D and 1,25(OH)_2_vitamin D increased significantly in the active treated group as compared to the placebo group (186% versus 14% (P<0.001) and 28% versus – 8% (P<0.001)). No change was observed in urinary calcium excretion in the active group compared to the placebo group (P = 0.891). Fibroblast growth factor 23 increased significantly by 10% (P<0.018) in the active group. However, there was no difference in changes in FGF23 between treatment groups (P = 0.457).

**Conclusion:**

High dose cholecalciferol significantly increases 25(OH)vitamin D and 1,25(OH)_2_vitamin D levels compared to placebo. No changes in urinary calcium excretion or other measured components of the mineral metabolism were found between groups.

**Trial Registration:**

ClinicalTrials.gov NCT00952562.

## Introduction

Low serum levels of 25(OH)vitamin D are frequently observed in healthy subjects [1], [2]. The definition of an optimal vitamin D level in different types of patients is currently under discussion [3]. When 25(OH)vitamin D concentrations decrease below 75 nmol/L (30 ng/ml), the parathyroid hormone levels (PTH) begin to increase [4], [5]. Keeping PTH inside the normal reference range is considered beneficial for bone, and serum 25(OH)vitamin D concentrations >75–100 nmol/L (>30–40 ng/ml) may be optimal for bone health [6].

Vitamin D deficiency is associated with serious chronic diseases, including autoimmune, infectious and cardiovascular diseases as well as cancer [7]–[9]. High serum concentrations of 25(OH)vitamin D are associated with increased bone mineral density (BMD), prevention of fractures, reduced fall risk, increased lower-extremity function and low incidence of colon cancer [7]. Improving vitamin D status could therefore be of potential health benefit. A large amount of vitamin D supplements are bought “over-the counter” by healthy people. However, the safety and possible adverse reactions of high intake of cholecalciferol in the general population has to be considered. Indeed, Melamed et al found a U shaped relation between survival and level of 25(OH)vitamin D in the general population, questioning the safety of high 25(OH)vitamin D levels [10].

Kidney stone disease typically presents between the ages of 20 and 60 years. Most recurrent kidney stone formers do not have a recognized underlying cause. High urine calcium is the single most common abnormality of urine chemistry in recurrent stone formers [11] indicating that hypercalciuria is an important risk factor for development of kidney stones.

Little is known about high dose vitamin D supplements and the risk of hypercalciuria in healthy people. Kidney stone disease is more prevalent in hot climates [12]. Whether this is due to increased sunlight exposure causing increased vitamin D synthesis is uncertain.

Vitamin D intoxication is traditionally considered related to hypercalcemia. It presents with nausea, dehydration and lethargy. Former research indicate that vitamin D intoxication in terms of hypercalcemia is generally not observed unless 25(OH)vitamin D concentrations exceed 375–500 nmol/L [13], [14], indicating that vitamin D may be administered liberally. However, a positive calcium balance may induce hypercalciuria even before hypercalcemia develops. The development of hypercalciuria may increase the risk of kidney stone formation and ultimately in a deterioration of renal function [15].

The changes in mineral metabolism including the changes in urinary calcium excretion should be investigated in detail before recommending high daily doses of vitamin D to otherwise healthy people.

The aim of this study was to investigate if treatment with a daily dose of 3000 IU cholecalciferol in healthy Danish adults affects mineral metabolism, especially urinary calcium excretion.

## Materials and Methods

The protocol for this trial and supporting CONSORT checklist are available as supporting information; see Checklist S1 and Protocol S1.

### Subjects

The study population consisted of healthy men and women working at Roskilde University Hospital. The subjects were recruited through posters and information meetings at Roskilde University Hospital.

The inclusion criteria were: age >18 years and the presence of vitamin D insufficiency (s-25(OH)vitamin D≤50 nmol/L (20 ng/ml)). To avoid inclusion of patients with known calcium metabolic disturbances or patients on medication which could influence the calcium balance, the exclusion criteria were treatment with vitamin D analogues, antihypertensives, antidiabetics or calcimimetics; sarcoidosis; cancer; (on-going or less than 5 years before this trial); pancreatitis; malabsorption; pregnancy, unsafe contraception, nursing; former kidney stones; p-creatinine >120 µmol/L; ionized p-calcium >1.50 mmol/L; former admission due to alcohol related disease; consumption of intoxicating substances; known intolerance to the study medication.

### Design

**Figure 1:**
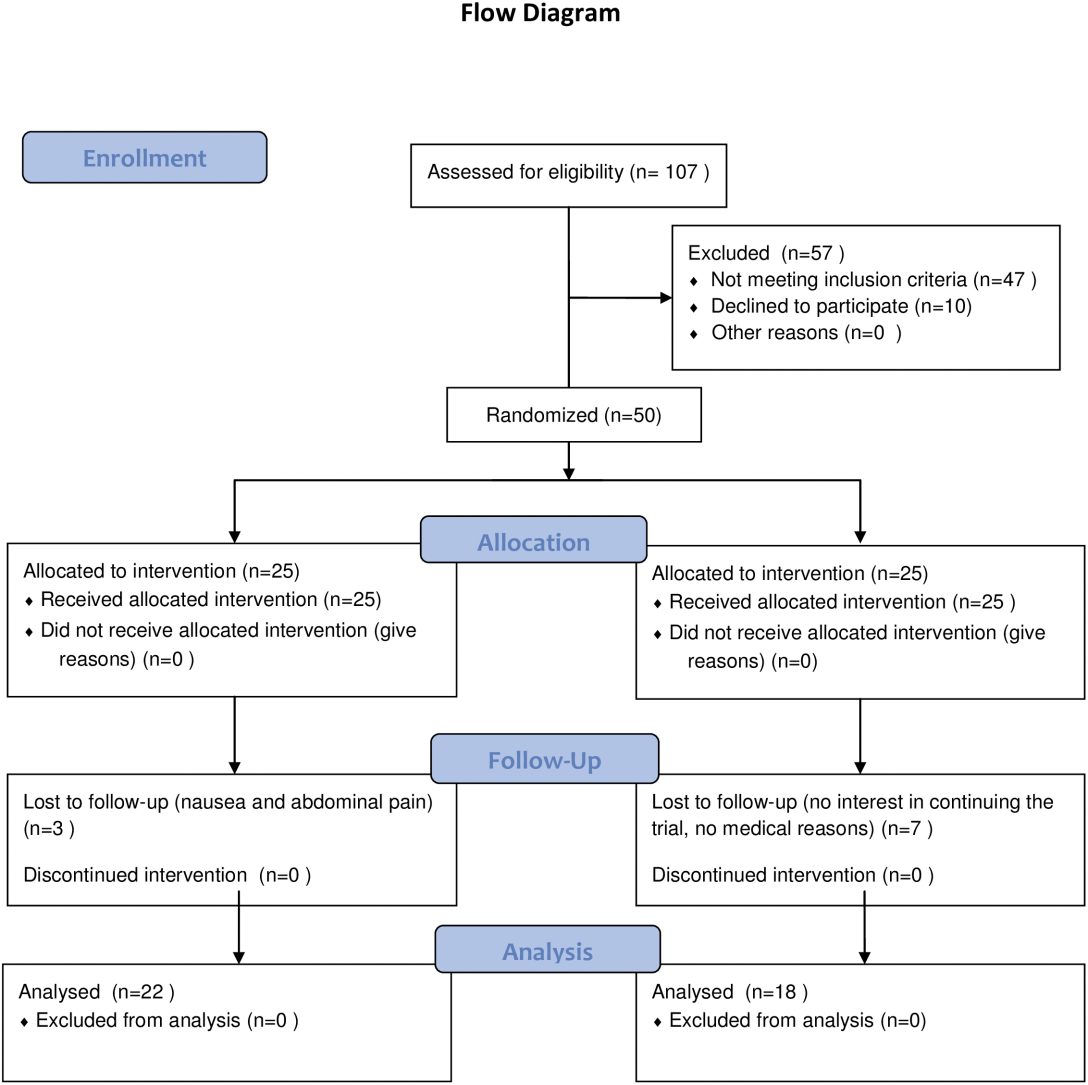
Flow diagram demonstrating participantś flow through the study.

In this double-blinded, placebo-controlled trial, subjects were randomized to receive a daily oral dose of 3000 IU of cholecalciferol (3 tablets of D3) or matching placebo tablets for 16 weeks (Figure 1). The active and placebo tablets were delivered in identical containers. A computer generated randomization list was used to pack the medication in consecutively numbered containers. The medication was given to the participants consecutively as they entered the study. The randomization list was kept by HCH until the study was closed. HCH was not involved in enrollment or assignment of participants to intervention during the study. Both participants and care providers were blinded during the study. The data analysis was performed unblinded.

The subjects were recruited during the winter December 2009 - January 2010 and December 2010 -January 2011, where vitamin D insufficiency is most prevalent in Denmark [2].

At study entry (week 0) and study end (week 16) a physical examination, assessment of the medical history, blood specimen and 24-hour urine sample was performed. Safety visits at week 4, 8 and 12 registered adverse events and measured plasma ionized calcium and phosphate. The subjects were advised to maintain their usual diets and to avoid taking additional vitamin or mineral supplements, licorice, herbs tablets, herbs tea or fish oil. The subjects were advised to take the tablets together with a meal. The cholecalciferol and placebo tablets were identical in appearance and contained the same excipients. Every subject received four bottles each containing 100 tablets. Compliance was assessed by counting the number of tablets at the final visit.

The primary endpoint of the study was changes in 24-hour calcium excretion in the urine (dU-calcium). No change in trial outcome was made after the trial commenced.). The dU-calcium in untreated osteoporotic patients is 5.0±2.0 mmol [16]. Hypercalciuria is defined as dU-calcium of 7.0 mmol [17]. The variation between individuals was considered to be more pronounced than within individuals, and a variation of 2.0 mmol was used for calculation of the sample size. To detect a difference of 2 mmol in dU-calcium with a statistical power of 87% and a significance level of 5% the sample size of each group was calculated to be 20. Each drop out was replaced by including and randomizing a new participant until 40 participants had completed the study. No interim analysis was performed. The study stopped at the final patient final visit. Secondary endpoints were dU-calcium >8.1 mmol (upper reference range at Frederiksberg Biochemical Department, Denmark), measurement of serum 25(OH)vitamin D, 1,25(OH)_2_vitamin D, total and bone specific alkaline phosphatase, plasma PTH, ionized calcium, magnesium, phosphate, fibroblast growth factor 23 (FGF23) and 24-hour phosphate excretion in the urine (dU-phosphate).

### Ethics statement

The study was performed at The Department of Nephrology, Roskilde University Hospital, Denmark. All participants signed informed consent forms. The study is in compliance with the Helsinki Declaration II of 1975, revised 1983 and was approved August 17 2009 by the Danish National Committee on Biomedical Research Ethics (SJ-135) and the Danish Data Protection Agency. The trial was registered at ClinicalTrials.gov (NCT00952562). The URL: http://clinicaltrials.gov/ct2/results?term=NCT00952562&Search=Search.

### Laboratory analysis

Blood samples for measurement of 25(OH)vitamin D, 1,25(OH)_2_vitamin D, fibroblast growth factor 23 (FGF23), total and bone specific alkaline phosphatase were collected and stored at −80°C. The analysis was performed in the same assay to eliminate inter-assay variation. Blood samples were taken at week 0 and week 16 in a fasting state (minimum 8 hours). Plasma ionized calcium, PTH, phosphate, creatinine and magnesium were measured on a routine basis by the same standardized analysis at the local laboratory.

Total and bone specific alkaline phosphatase were measured in serum by an immunoassay (METRA BAP EIA KIT, QUIDEL CORPORATION, San Diego, CA, USA). The coefficients of variation (CV) of the assay were 4.2% and 6.7% at concentrations of 15 and 65 U/L, respectively.

FGF23 was measured in plasma by a sandwich enzyme-linked immunosorbent assay (Kainos Laboratories Inc., Tokyo, Japan). This assay detects only the biologically active intact FGF23. The intra- and inter assay CV was less than 5.0%.

Serum 25(OH)vitamin D was determined by DiaSorin LIAISON, Sundbyberg, Sweden. This direct competitive chemiluminescence immunoassay determines both 25(OH)vitamin D2 and 25(OH)vitamin D3. Precision ranges (CV%) were: within run (2.8–8.1%) and total precision (7.3–17.5%). Measuring range was 4.0 to 150 ng/mL.

Serum 1,25(OH)_2_vitamin D was determined by a radioimmunoassay (Gamm-B1, 25-Dihydroxyvitamin D, Immunodiagnostic Systems [IDS], Ltd., Boldon, England). The CV of the assay was between 6.8% and 14.0% in the range from 16 to 220 pmol/L.

Urine was collected during 24 hours in 2.5 liter containers. Separate collection of calcium/protein and phosphate was done on two consecutive days. The containers used for phosphate collection were added 2.5% hydrochloric acid. Calcium and protein was collected in clean containers. Diet registration was documented in a diary. Diet diary was performed three days prior to the 24-hour urine collection period at week 0 and the patients were instructed to follow the same dietary regime three days prior to the 24-hour urine collection at week 16.

### Statistical analysis

The biostatistical evaluation was carried out using SAS JMP for Windows (version 9.0.2). Continuous data were described as mean (SD) and for differences the mean (95% confidence interval (CI)) if data were normally distributed, and as median (interquartile range, 25–75 th percentile) if data were not normally distributed.

When comparing the two groups, data were analyzed by an unpaired T-test. If a deviation from normality was found a Mann Whitney test was applied. FGF23 was logarithmically transformed.

When analyzing changes in each group during the course of the study a paired T-test was used (matched pairs). In cases where deviation from normality was found a Wilcoxon signed rank test was used.

Bonferroni correction was applied to account for multiple testing, and the threshold for statistical significance was P<4.55×10^−3^(0.05/11).

Linear regression analyses was performed between the variables that did change significantly between the groups during the course of the study (change in 1,25(OH)_2_vitamin D vs. baseline 25(OH)vitamin D)_._ The Z-test was applied to investigate whether the correlation was different between the two groups (the cholecalciferol group (β_1_) and the placebo group (β_2_)).

Correlation between changes in 25(OH)vitamin D and changes in PTH, s-phosphate, urinary phosphate excretion, ionized calcium, magnesium, and total and bone specific alkaline phosphatase were described by Pearsons correlation coefficient.

Multiple imputation was performed by the procedures proc MI and proc MIAnalyze in SAS version 9.3. Imputed data were generated by regression of treatment group, urinary calcium, dU-calcium and 1,25(OH)_2_vitamin D. Twenty imputed dataset were generated and the results of these were combined [18].

All tests were two-sided tests (α = 0.05).

## Results

### Subjects

Of the 107 screened subjects 47 were excluded due to serum 25(OH)vitamin D >50 nmol/L (20 ng/ml) and 10 subjects declined to participate. In total 50 subjects (25 females and 25 males) were randomized in order to reach 40 subjects completing the study (Figure 1). All subjects were caucasian.

We observed a small, but significant difference in baseline ionized calcium between the groups (P = 0.006). All other baseline parameters were similar between the two groups. The baseline characteristics of the subjects completing the study were not different from the baseline characteristics of the randomized subjects (Table 1).

**Table 1:** Baseline data of participants who completed the study.

	Cholecalciferol (N = 22)	Placebo (N = 18)
Age (years)	42.8 (9.2)	47.0 (8.1)
Sex (female)	11 (48%)	6 (33%)
Height (cm)	168 (19)	175 (8)
Weight (kg)	74.6 (14.0)	77.7 (14.6)
Systolic blood pressure (mmHg)	118 (11)	118 (10)
Diastolic blood pressure (mmHg)	72 (7)	73 (6)
Creatinine (µmol/l)	76 (12)	80 (11)

Data are means (SD) or numbers (%).

Based on tablet count full compliance was achieved.

### Adverse events and exclusion

During the study, 2 subjects in the cholecalciferol treated group refused to continue due to nausea and slight abdominal pain (one at week 6 and one at week 8). Both of them had normal serum calcium and phosphate levels at the safety visits week 4 and 8, respectively. One subject discontinued the study shortly after week 0 because of pregnancy. In the placebo group 1 subject discontinued due to exanthema in connection with NSAID treatment (week 6) and 6 subjects refused to continue the study due to lack of interest and time (Figure 1).

### Mineral metabolism

**Figure 2:**
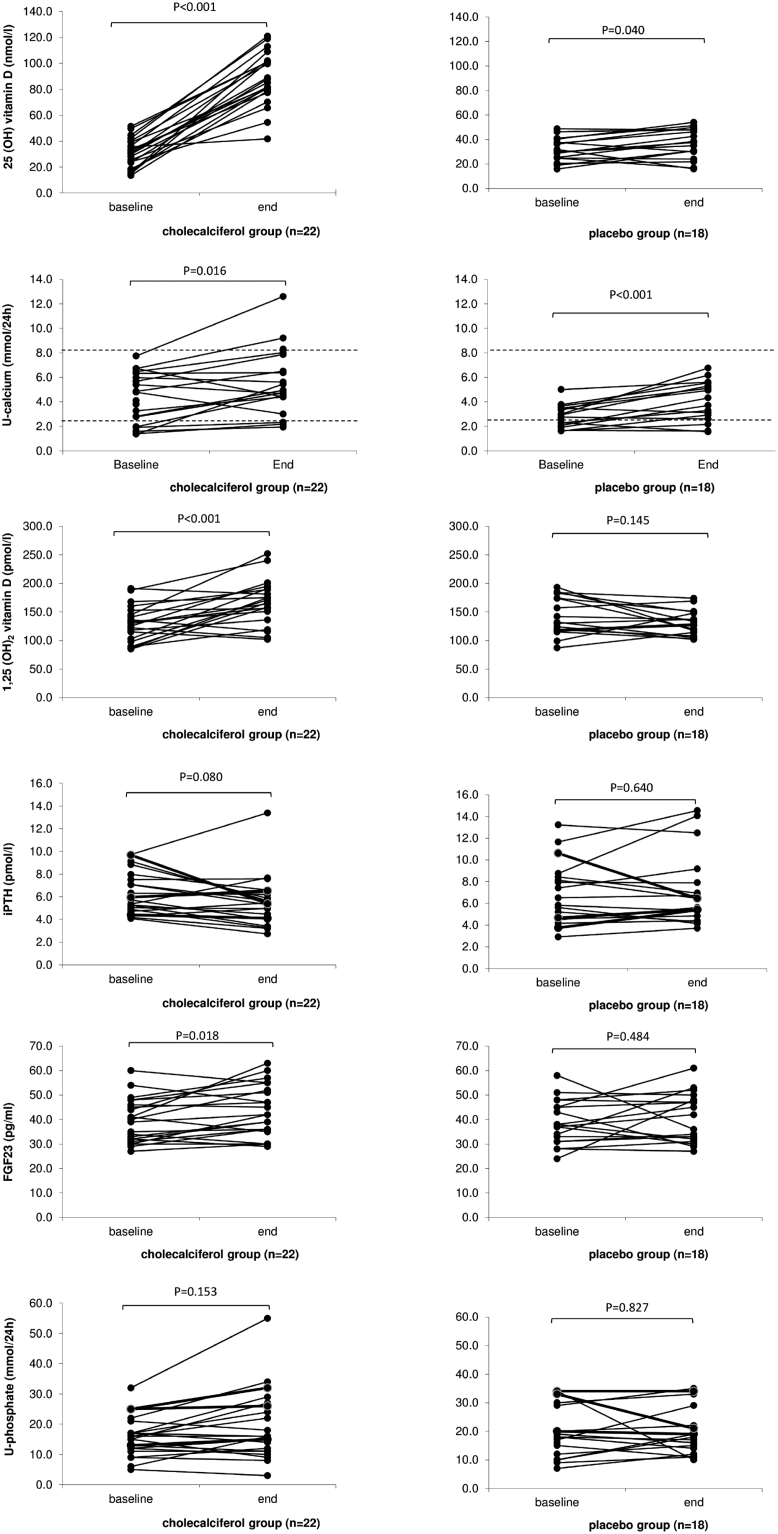
25(OH)vitamin D, urinary calcium excretion, 1,25(OH)_2_vitamin D, PTH, FGF23 and urinary phosphate excretion at baseline and after 16 weeks of treatment with cholecalciferol 3000 IU/day or placebo. Urinary calcium excretion: The laboratory reference interval (2.5–8.1 mmol/24 hour) is illustrated with dotted lines.

Baseline and final laboratory values of the subjects are presented in Table 2 and Figure 2.

**Table 2:** Baseline and final laboratory values of the subjects.

	Cholecalciferol (n = 22)		Comparison of changeswithin group. *Mean* *difference (95% {CI})* *P-value*	Placebo (n = 18)		Comparison of changeswithin group. *Mean* *difference (95% {CI})* *P-value*	Comparison of changesbetween groups. *Difference* *(95% {CI}) P-value*
	Baseline*Mean (SD)*	16 weeks*Mean (SD)*		Baseline*Mean (SD)*	16 weeksmean *(SD)*		
25 (OH) D nmol/l	30.86 (11.08)	88.25 (20.02)	57.39 {48.78; 66.00}P<0.0001*	32.42 (9.81)	37.11 (12.19)	4.69 {0.23; 9.14} P<0.040*	−52,70 {−62.19; −43.22}P<0.0001*
1,25 (OH)_2_ D pmol/l	129.23 (30.86)	164.77 (39.07)	35.55 {18.03; 53.06}P<0.0004*	141.67 (33.16)	130.61 (21.54)	−11.06 {−26.34; 4.23} P = 0.145	−46.60 {−69.09; −24.11}P<0.0002*
PTH pmol/l	6.29 (1.83)	5.58 (2.22)	−0.71 {−1.5; 0.09}P = 0.080	6.88 (2.87)	7.11 (3.34)	0.23 {−0.78; 1.24} P = 0.640	0.94 {−0.31; 2.18}P = 0.136
Ion ca mmol/l	1.21 (range 1.15–1.25)	1.21 (range 1.16–1.25)	0 P = 0.832	1.23(range 1.19–1.30)	1.24(range 1.11–1.27)	0.01 P = 0.601	0.01 P = 0.671
Mg mmol/l	0.845 (range 0.71–1.17)	0.84 (range 0.74–0.92)	0.005 P = 0.423	0.86(range 0.69–0.96)	0.805(range 0.74–0.93)	−0.055 P = 0.966	0.050 P = 0.540
Phosphate mmol/l	1.23 (0.19)	1.12 (0.17)	−0.01 {0.08; 0.06}P = 0.761	1.03 (0.15)	1.08 (0.14)	0.04 {−0.04; 0.13} P = 0.270	0.05 {−0.05; 0.16}P = 0.290
T. alk. phosp U/l	61.04 (20.65)	57.41 (19.98)	−3.63 {−0.86; −6.40}P<0.013	65.11 (17.53)	64.06 (17.11)	−1.06 {−3.82; 1.71} P = 0.432	2.58 {−1.21; 6.37}P = 0.177
B. spec. alk. phosp U/l	21.27 (7.45)	22.58 (6.53)	1.31 {−1.61; 4.23}P = 0.362	22.81 (7.81)	22.90 (6.71)	0.09 {−1.26; 1.45} P = 0.888	−1.22 {−4.37; 1.94}P = 0.437
FGF23 pg/ml	39.45 (8.96)	43.45 (10.46)	4 {0.77; 7.23}P<0.018*	38.72 (9.14)	40.56 (10.07)	1.83 {−3.58; 7.25} P = 0.484	−2.17 {−8.31; 3.97}P = 0.476
U phosp mmol/24 hour	27.30 (7.85)	31.77 (13.33)	4.47 {−1.8; 10.74}P = 0.153	32.49 (12.71)	33.73 (17.14)	1.24 {−10.60; 13.07} P = 0.827	−3.24 {−16.28; 9.81}P = 0.613
U ca mmol/24 hour	4.32 (2.17)	5.48 (2.69)	1.17 {0.25; 2.08}P<0.016*	2.80 (0.96)	4.05 (1.64)	1.24 {0.58; 1.90} P<0.001*	0.08 {−1.02; 1.17}P = 0.889
U protein mg/24 hour	8.57 (4.04)	15.38 (14.01)	6.18 {−0.84; 14.47}P = 0.08	10.33 (11.84)	13.03 (11.89)	2.72 {−4.43; 9.84} P = 0.43	−4.10 {−14.13; 5.92}P = 0.41

25 (OH) D, s-25-hydroxyvitamin D; 1,25 (OH)_2_D, s- 1,25 dihydroxyvitamin D; PTH, p-parathyroid hormone; Ion Ca, p-ionized calcium; Mg, p-magnesium; phosphate, p-phosphate; T. alk. phosp, s- total alkaline phosphatase; B. spec. alk. phosp, s-Bone specific alkaline phosphatase; FGF23, p- fibroblast growth factor 23; U phosp, urinary phoshate excretion; U Ca, urinary calcium excretion.

n = number of subjects in each group. SD standard deviation. p = plasma, s = serum.

Within group comparisons was performed by Matched Pairs Test. When not normally distributed Wilcoxon signed rank test was applied (Ion Ca and mg), and variable described by median and range (0–100%).

* = P<0.05∶16 week measurement was significantly different from 0 week.

Between groups comparisons was performed by unpaired T-test. Difference between placebo and cholecalciferol group (mean difference). When unable to achieve normal distribution Mann- Whitney’s test was used (Ion Ca and Mg).

* = P<0.05: cholecalciferol group significantly different from the placebo group.

**Figure 3:**
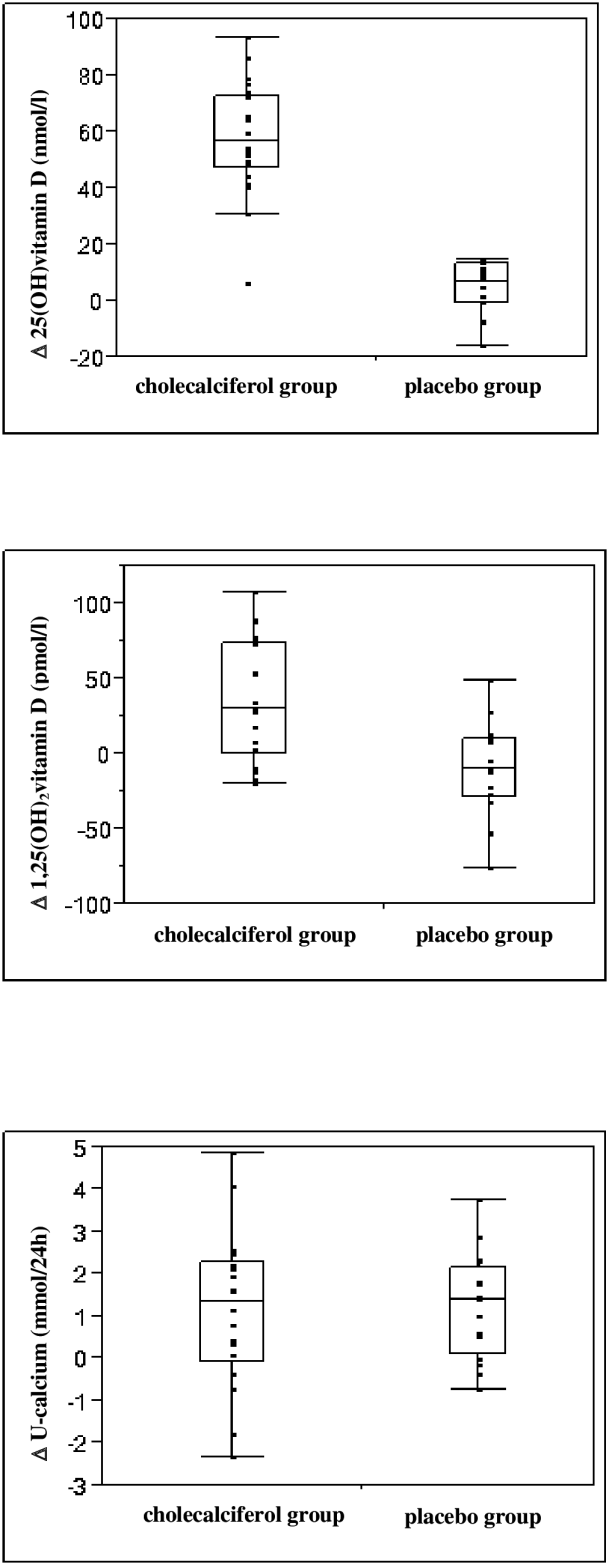
Box plots demonstrating changes in 25(OH)vitamin D, 1,25(OH)_2_vitamin D and urinary calcium excretion in the cholecalciferol group and placebo group during 16 weeks of treatment with cholecalciferol 3000 IU/day or placebo.

Both 25(OH)vitamin D and 1,25(OH)_2_vitamin D increased significantly in the cholecalciferol treated group as compared to the placebo group (186% vs. 14%; P<0.0001 and 28% vs. −8%; P<0.0002, respectively) (Figure 3). Statistical significance was preserved after Bonferroni correction for multiple comparisons (25(OH)vitamin D; P<0.001 and 1,25(OH)_2_ vitamin D;P<0.002).

In the cholecalciferol treated group 18 subjects (82%) achieved a serum concentration of 25(OH)vitamin D above 75 nmol/L. These 18 subjects had a non statistically significant higher baseline value of 25(OH)vitamin D (32.0±11.7 vs. 26.0±7.1; P = 0.223). No subjects in the placebo group had 25(OH)vitamin D concentrations above 75 nmol/L at any time during the study.

FGF23 increased significantly by 10% in the cholecalciferol treated group (P<0.018). FGF23 values did not change in the placebo group (P = 0.484). There was no correlation between changes in FGF23 and urinary phosphate excretion (β = −0.13; P = 0.15).

Total alkaline phosphatase showed a significant decrease (6%) in the cholecalciferol group (P<0.013). In contrast, bone specific alkaline phosphatase did not change significantly during the course of this study in any group.

No significantly difference in changes in dU-calcium was observed between the groups (P = 0.891) (Table 2). Due to the presence of 10 dropped out subjects, a sensitivity analysis was performed. This did not find a significant difference in dU-calcium between treatments groups (P = 0.688). However, dU-calcium increased significant in both the cholecalciferol treated group (27%) and the placebo group (44%) (P<0.016 and P<0.001 respectively) (Table 2, Figure 2 and 3). In 3 subjects in the cholecalciferol treated group the dU-calcium at week 16 increased above the upper reference range of 8.1 mmol/24 hour (Figure 2). Unfortunately, in one of these subjects the baseline dU-calcium value was not available. A larger increase in dU-calcium was found among the subjects with a final dU-calcium >8.1 mmol (2 subjects: 2.49 and 4.86 mmol) compared to the other subjects in the cholecalciferol group (mean 3.67 (1.68) vs. 0.85(1.66)). We did not observe any difference in baseline or delta values of 25(OH)vitamin D, 1,25(OH)_2_vitamin D and FGF23 in the 3 subjects with dU-calcium above upper reference at 16 week compared to the other subjects. A much larger standard deviation in baseline dU-calcium values was found in the cholecalciferol group compared to placebo (2.17 vs.0.96). We found subjects with a baseline value of dU-calcium close to upper reference interval (8.1 mmol), but we did not find any correlation between baseline 25(OH)vitamin D and baseline dU-calcium (β = 0.02, P = 0.584). Surprisingly, 4 subjects in the cholecalciferol group showed a decrease in dU-calcium (mean = −1.29 (0,91)). All 4 presented an increase in 25(OH)vitamin D at week 16 (mean 58.38 (14.42)), and did not differ from other cholecalciferol treated subjects in terms of changes in total alkaline phosphatase, bone specific alkaline phosphatase and 1,25(OH)_2_vitamin D. All subjects in the placebo group showed dU-calcium values within the laboratory reference interval both at baseline and at week 16 (Figure 2).

The changes in urinary calcium did not differ between subjects reaching 25(OH)vitamin D levels above vs. below 75 nmol/L (P = 0.50).

As shown in Table 2 all other laboratory values stayed within the normal reference range and did not change significantly between the two groups. Also after Bonferroni correction the values did not change significantly between the two groups.

**Figure 4:**
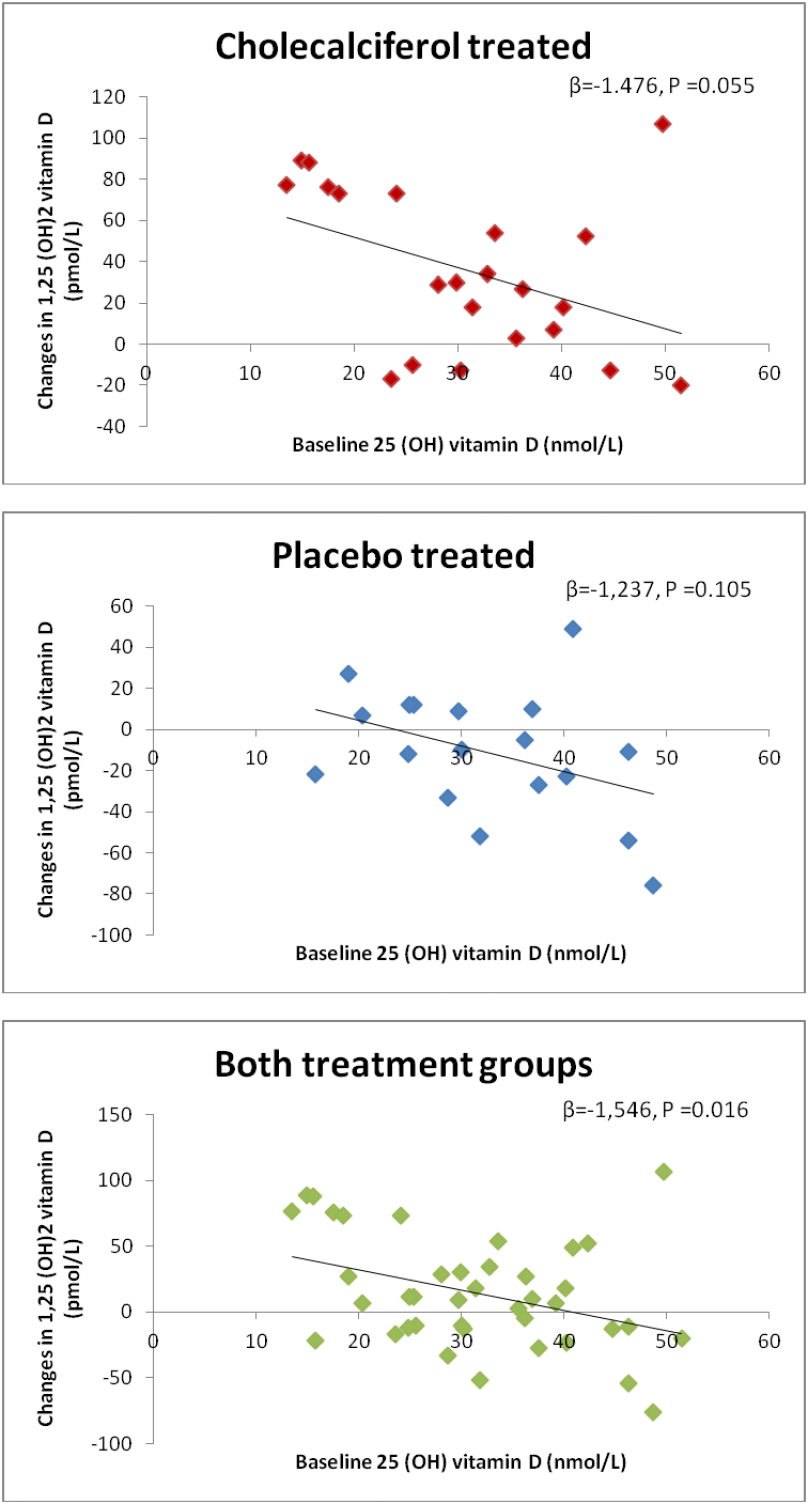
Changes in 1,25(OH)_2_vitamin D according to baseline level of 25(OH)vitamin D in the cholecalciferol group, the placebo group and both treatment groups. Changes in 1,25(OH)_2_vitamin D are depending on the baseline level of 25(OH)vitamin D β = linear regression coefficient.

There was a positive but not statistically significant correlation between changes in 25(OH)vitamin D and 1,25 (OH)_2_ vitamin D in both groups (β = 0.483, P<0.287 and β = 1.440, P<0.083; active and placebo respectively). A negative non significant correlation was found between baseline values of 25(OH)vitamin D and the changes in 1,25(OH)_2_vitamin D in the cholecalciferol group and the placebo group. In pooled analysis of both groups we found this correlation statistically significant (Figure 4). We did not find any significantly different correlation between the cholecalciferol group and the placebo group (Z^2^ = 0.055<3.84).

The correlation between changes in 25(OH)vitamin D levels and changes in PTH, s-phosphate, urinary phosphate excretion, ionized calcium, magnesium, total and bone specific alkaline phosphatase was also examined. Only the correlation between changes in 25 (OH)vitamin D and urinary phosphate excretion was found to be significant in the cholecalciferol treated group (β = −0.324, P<0.033).

Urinary protein excretion did not change in any group (Table 2). There was no association between changes in 25(OH)vitamin D and urinary protein excretion (β = 0.05, P = 0.59).

## Discussion

The risk of kidney stone formation increases with increasing urinary calcium excretion and patients treated with vitamin D may have increased risk of kidney stone formation [19]. In this randomized, double-blinded, placebo-controlled trial, we found no difference in changes in urinary calcium excretion during 16 weeks of treatment with 3000 IU a day of cholecalciferol compared to placebo.

Epidemiologic studies have reported associations between urinary calcium excretion and serum levels of 25(OH)vitamin D and 1,25(OH)_2_vitamin D [20], [21]. Although intervention studies have demonstrated conflicting results regarding vitamin D and the risk of hypercalciuria and kidney stone formation.

In postmenopausal women a 12 to 33% increase in urinary calcium excretion was found during treatment with variable doses of cholecalciferol compared to placebo [22]. In the Women Health Initiative Clinical trial a large randomized trial of around 36,000 postmenopausal women a 17% increased risk of kidney stone formation was found in women receiving 400 IU cholecalciferol and 1000 mg calcium per day compared to placebo. Whether this increase was due to calcium, vitamin D or both is difficult to distinguish, and the study did not report data on urinary calcium excretion [23]. A unique bone metabolism in postmenopausal women may explain higher calciuria after vitamin D therapy, and it is unknown whether these results are applicable to healthy subjects.

Isidro et al demonstrated that during 12 months of treatment with calcidiol (max dose 960 IU) mean urinary calcium excretion increased significantly [24]. However, the participants in this study were patients with asymptomatic primary hyperparathyroidism. These subjects are probably not comparable to healthy subjects with normal bone metabolism.

Our findings in healthy individuals are supported by two other trials: In a prospective, yet not placebo-controlled study by Vieth et al, healthy men and women were randomly assigned to receive either 25 µg or 100 µg vitamin D_3_ per day for 2–5 months. Serum calcium and urinary calcium excretion did not change significantly at either dose during this study [6]. In a 6 months prospective, randomized double-blinded placebo-controlled study aiming to determine the intake of vitamin D needed to raise 25(OH)vitamin D>75 nmol/l (mean daily dose 3440 IU) a few subjects had hypercalciuria, but no difference in the number of active and placebo subjects evidencing hypercalciuria [25]. Our trial, as well as the trials mentioned above, are small, and there may be minor changes in urinary calcium excretion that are not detected due to the limited sample size. We found an increase in urinary calcium excretion outside the laboratory reference interval, in three individuals in the cholecalciferol treated group. The absolute increase in dU-calcium in these individuals was higher than in the remaining group. Furthermore, 4 subjects in the cholecalciferol group showed a decline in dU-calcium, despite an increase in 25(OH)vitamin D. These findings indicate that the sensitivity to the calciuric effect of vitamin D may differ between individuals. This variation in sensitivity to the calciuric effect of vitamin D and the clinical consequences remains to be further explored, especially taking other risk factors for developing kidney stones into account including life style and dietary factors such as fluid intake, high salt intake, low calcium intake, hypocitraturia, high animal protein intake and hyperoxaluria [11]. A recent uncontrolled trial in kidney stone formers found no significant increase in calciuria during eight weeks of ergocalciferol 50000 IU per week. However, 11 subjects had an increase in dU-calcium above 20 mg/d [26]. This supports our thesis that some individuals might be more sensitive to the calciuric effect of vitamin D. It is unclear whether the magnitude of increase in urinary calcium excretion can be attributable to dietary changes. Although, we made an effort to standardize the dietary intake prior to the baseline and the final visit, there still may be some variation in the calcium intake influencing the urinary calcium excretion.

The reference interval for dU-calcium applied in the present study is based on a normal population material from before 2007. High dose of vitamin D supplements were not recommended at this time. A high frequency of vitamin D supplementation in the general population may increase the level of the reference interval. Whether, an increased level of dU-calcium in the general population would lead to increased incidence of kidney stone formation is unknown. However, it is well known that hypercalciuria is present in 40 to 60% of patients with calcium containing stones [27], and it appears plausible that an increase in dU-calcium may increase the incidence of kidney stone formation.

A great variability in the dU-calcium baseline value was found among the healthy Danish subjects with vitamin D deficiency in the present study. We even found subjects with a baseline value close to upper reference limit (8.1 mmol/24 h). We were surprised not to find any correlation between baseline 25(OH)vitamin D and baseline dU-calcium among our subjects. The reason may be individual differences in calciuric response to vitamin D as mentioned above.

The levels of 25(OH)vitamin D and 1,25(OH)_2_vitamin D increased significantly in the cholecalciferol treated group compared to the placebo group. Oral vitamin D is rapidly absorbed from the intestine and 25-hydroxylated in the liver. This process is not rate limited [9]. An increase in serum 25(OH)vitamin D is therefore expected. In case of vitamin D deficiency 25(OH)vitamin D is further hydroxylated mainly in the kidney by the enzyme 1-alpha hydroxylase to the active metabolite 1,25(OH)_2_vitamin D. In the present study the inclusion criteria was vitamin D deficiency. Although, initial serum 1,25(OH)_2_vitamin D value was in the normal range we observed a significant increase. This increase in 1,25(OH)_2_vitamin D is consistent with previous studies [28]–[30]. Levels of 1,25(OH)_2_vitamin D are regulated by a negative feed-back of 1,25(OH)_2_vitamin D on 1α-hydroxylase and a positive regulation of 24-hydroxylase [31], [32]. The observed increase in 1,25(OH)_2_vitamin D may indicate a deficiency that is corrected when the levels of the precursor 25(OH)vitamin D are increased. Indeed, we found a negative correlation between baseline levels of 25(OH)vitamin D and the increase in 1,25(OH)_2_vitamin D. The small changes in the placebo group confirms that high levels of 25(OH)vitamin D stimulates a decrease in 1,25(OH)_2_vitamin D and low levels stimulates an increase in 1,25(OH)_2_vitamin D. These changes are more pronounced in the intervention group where the level of 25(OH)vitamin D are increased, and when pooling both groups there is enough power to demonstrate a significant correlation.

The reason for the increase in 25(OH)vitamin D in the placebo group remains uncertain. We did not collect data on travel to areas with more sun exposure or use of solarium. Participation in the study could lead to changes in the dietary habits in the participants. The increase in 25(OH)vitamin D in the placebo group may explain the significant increase in the dU-calcium in the placebo group, and may be the reason why we did not find a difference between treatment groups in dU-calcium.

We found a significant increase in FGF23 in patients treated with cholecalciferol, although no significant difference in FGF23 changes was found between groups. This may be due to the small sample size, or the increase in 25(OH)vitamin D in the placebo group.

This is to our knowledge the first randomized controlled study to explore the changes in FGF23 during treatment with cholecalciferol in healthy men and women. This supports the increase in FGF23 after replacement of vitamin D deficiency, which has been demonstrated after substitution with 50000 IU ergocalciferol [33] and the increase in FGF23 observed in an uncontrolled trial of cholecalciferol substitution in women only [34].

The influence of cholecalciferol and active vitamin D metabolites on FGF23 has also been described in patients with chronic kidney disease. Both 1,25(OH)_2_vitamin D, other 1α-hydroxylated vitamin D analogues [35]–[37] and cholecalciferol in doses of 40000 IU per week increase the circulating levels of FGF23 [38] in patients with chronic kidney disease. The response to vitamin D may differ between healthy and uremic patients. Indeed in bone cultures derived from uremic rats the response to calcitriol was found to be more pronounced than in normal rats, probably due to up-regulation of the vitamin D receptor (VDR). The induction of the VDR that occurs in the calvaria of the uremic rats differs from the major loss of VDR in other regulators of mineral metabolism as the parathyroid glands or the kidney during uremia [39].

FGF23 inhibits the renal phosphate reabsorption and forms a counter regulation of the vitamin D induced increase in intestinal phosphate reabsorption. However, in this study we did not detect any difference in the urinary phosphate excretion or circulating phosphate levels. Whether changes in FGF23 are involved in kidney stone formation is unexplored. Increasing levels of FGF23 are associated with increased cardiovascular disease and mortality in the community [40]–[45]. Whether the observed increase in FGF23 in the present study is of clinical importance is unknown.

There are some limitations to the present study. Based on the common use of vitamin D supplements among otherwise healthy subjects we found it necessary to investigate possible side effects. However, the results of this study will be less applicable to patients with chronic illness or diseases affecting bone metabolism. Second, the sample size was calculated to be 20 in each arm. Unfortunately, 22 completed the study in the cholecalciferol group and only 18 in the placebo group. This decreases the power to detect a relevant difference between groups. Third, this study was powered to detect a difference of dU calcium of 2 mmol. Differences less than dU calcium 2 mmol may exist, which the present study would not have the power to detect. Fourth, a larger variation in baseline urinary calcium was found in the active treated group as compared to placebo group. Fifth, we only measured calcium in one 24-hour urine sample before and after intervention. Two 24-hour samples would be optimal. However, we sought to standardize the dietary intake for each individual in order to minimize the day to day variation. Sixth, this study was performed in wintertime. The results during summer may differ, as 25(OH)vitamin D is known to increase due to sun exposure during this period of the year in the northern hemisphere [46].

## Conclusion

In the present randomized controlled trial, we found that 3000 IU of cholecalciferol per day during 16 weeks increased vitamin D levels both in terms of 25(OH)vitamin D and 1,25(OH)_2_vitamin D compared to placebo. FGF23 increased significantly in the cholecalciferol treated group although not statistically significant from placebo.

There was no significant change in urinary calcium excretion between the groups, although in 3 individuals urinary calcium excretion increased above the reference interval in the cholecalciferol treated group.

We speculate whether some individuals could be more sensitive to cholecalciferol treatment, concerning urinary calcium excretion.

## Supporting Information

Protocol S1 **Trial protocol.**
(DOC)

Checklist S1 **CONSORT checklist.**
(DOC)
