# Supplementary material for: Effects of High Doses of Cholecalciferol in Normal Subjects: A Randomized Double-Blinded, Placebo-Controlled Trial
Source: PLoS One. 2014 Aug 28;9(8):e102965. doi: 10.1371/journal.pone.0102965 (PMC4148309; doi:10.1371/journal.pone.0102965)
Supplement: Protocol S1 — Trial protocol. (DOC) [file pone.0102965.s001.doc]

# Mineralmetabolismen, blodtryk og pulsbølgemåling ved

# D vitamin behandling

Ditte Hansen, læge, ph.d. studerende

Investigator og ansvarlig overfor videnskabsetisk komite og datatilsynet

Niels Erik Frandsen, overlæge

Investigator

Knud Rasmussen, overlæge, dr.med.

Investigator

Lisbet Brandi, ledende overlæge, dr.med.

Investigator

Medicinsk afdeling

Roskilde Sygehus

Køgevej 7-13

4000 Roskilde

Hans Christan Høck

Klinikchef, PhD

Speciallæge i intern medicin og endokrinologi

Center for Clinical and Basic Research

Ansvarlig for randomisering pakning og etikettering af studiemedicin samt opbevaring af randomiseringslisten

**Baggrund**

Flere patient kategorier får påvist lavt niveau af plasma D vitamin, eventuelt ledsaget af forstyrrelser i mineralmetabolismen. Disse patienter tilrådes tilskud med D vitamin. Det er overraskende at selv raske personer kan have et lavt plasma vitamin D niveau, dette til trods for normal kostindtag evt suppleret med vitamintabletter som indeholder vitamin D [1,2].

Det er omdiskuteret hvor grænsen for normalt plasma D vitamin niveau bør placeres [3]. Traditionelt har man accepteret plasma 25 hydroxyvitamin D > 50nmol/l med baggrund i det nødvendige niveau for at supprimere produktionen af parathyroideahormon.

Gennem de sidste år har der været en tendens til stile mod at placere grænsen højere (80 nmol/l) med baggrund i nyere studier som har fundet an association mellem høj plasma 25 hydroxyvitamin D og en række tilstande som høj knogletæthed, forebyggelse af frakturer, nedsat faldtendens, øget muskelstyrke og lav forekomst af colon cancer [4]. Dette medfører et tilsvarende øget behov for D vitamin tilskud svarende til ≥ 1000 IU/dag [4,5]. Vitamin D_3_ (cholecalciferol) giver en mere stabil serum koncentration end vitamin D_2_ (ergocalciferol) og bør foretrækkes [6].

Tidligere undersøgelser hvor store doser D vitamin er anvendt har været kombinationsbehandlinger hos osteoporose patienter [7]. D vitamin mangel er associeret med forhøjet blodtryk og kartivhed [8,9]. Desuden har enkelte studier vist en påvirkning af blodtrykket ved D vitamin behandling [10,11]. D vitamin er kategoriseret som kosttilskud og ikke som lægemiddel. Dette betyder at der ikke er de samme godkendelseskrav som hvis det havde været et lægemiddel. Skal vi følge anbefalingen om øget plasma niveau af 25 hydroxyvitamin D må vi anbefale højdosis vitamin D behandling. Forinden bør det undersøges om denne behandling påvirker kroppen uhensigtsmæssigt f.eks. med så store ændringer i urin calcium og fosfat at dannelse af bl a nyresten kan blive en bivirkning.

**Formål**

Vi ønsker at undersøge hvorledes mineralmetabolismen påvirkes specielt i form af calcium og fosfat udskillelsen i urinen under behandling med de nu hyppigt anvendte høje doser cholecalciferol hos voksne raske forsøgspersoner uden tilskud af calcium. Undersøgelsen vil også belyse påvirkningen af kredsløbet idet vi måler blodtryk og udfører pulsbølgeanalyse.

**Perspektiv**

Da store doser D vitamin anbefales til i øvrigt raske mennesker med lavt plasma D vitamin niveau er det væsentligt at kende deres indvirkning på mineralmetabolismen. Specielt betydningen for nyrernes håndtering af calcium og fosfat er interessant hvis også nyresyge patienter skal tilbydes denne behandling i fremtiden.

**Inklusionskriterier**

1. alder: > 18 år,
2. D vitamin insufficiens (P-25OHD < 50 nmol/l)
3. skriftligt samtykke efter mundtlig og skriftlig information.

**Eksklusionskriterier**

1. Behandling med antihypertensiva, antidiabetika
2. sarkoidose
3. tidligere nyresten
4. indtagelse af 1α hydroxylerede præparater (Etalpha)
5. Plasma-kreatinin > 120 μmol/l,
6. ioniseret Plasma-Ca (middel af 2 målinger) > 1,50 mmol/l,
7. behandling med calcimimetica: Cinacalcet (Mimpara®),
8. aktiv malign lidelse (pågående behandling/kontrolbesøg eller diagnosticeret mindre end 5 år forud for aktuelle)
9. malabsorption, herunder ventrikel / tarmresektion
10. aktiv pancreatitis (P-amylase > 3 x øvre referenceinterval),
11. tidligere indlæggelse grundet alkoholrelateret sygdom,
12. indtagelse af euforiserende stoffer,
13. graviditet eller usikker antikonception eller ammende
14. allergi for et eller flere af benyttede indholdsstoffer.

**Rekruttering**

Patienterne rekrutteres ved opslag på Roskilde Sygehus, på offentlige institutioner i Roskilde og omegn såsom biblioteker og ved voksenuddannelsessteder. Opslagene vil indeholde telefonnummer på forsøgsansvarlig hvor der kan modtages flere oplysninger om projektet. Opslagene ophænges fra uge 36 2009 og inklusion påbegyndes 1. september 2009.

**Antal patienter**

Døgnurin udskillelsen af calcium hos ubehandlede osteoporose patienter er 5,0 ± 2,0 mmol/24timer [7]. Hypercalcuri er defineret som dU-calcium>0,1 mmol/kg/24 timer sv.t. 7 mmol/døgn for en voksen person på 70 kg [12]. En stigning i dU-calcium på 2 mmol er derfor en relevant minimal forskel at påvise.

Da variationen mellem individer må antages at være større end inden for individer anvendes SD 2,0 mmol/24 timer til en meget konservativ styrkeberegning.

Med en statistisk power på 90% og et 5% signifikansniveau vil den nødvendige størrelse af hver gruppe være 20 for at kunne detektere en forskel på 2 mmol i dU-calcium[13].

**Fertile kvinder**

Fertile kvinder, som inkluderes i forsøget, skal anvende svangerskabsforebyggende midler i hele forsøgsperioden.

Gravide og ammende kvinder kan ikke deltage i undersøgelsen.

**Effektparametre**

Primær

Calcium udskillelse i urinen. Den højeste af to døgnurinmålinger.

Sekundær

Døgnurin for creatinin, protein, fosfat, Na, K, urat, blod og pH, urin calcium/creatinin ratio

p-iPTH, p-ioniseret calcium, p-phosphat, p-magnesium, p-25 hydroxyvitamin D, p-1,25 dihydroxyvitamin D. p-triglycerid og p-cholesterol, p-FGF-23.

BT, pulsbølgeanalyse, døgnblodtryk

**Medicin**

Deltagerne vil blive randomiseret til 4 måneders (16 uger) behandling med enten kaps. cholecalciferol eller identisk placebo. Der gives vitamin D_3_ (cholecalciferol) 3000 IE peroralt en gang dagligt i hele perioden.

Cholecalciferol 3000 IU/dag

N=20

Placebo

N=20

Randomisering

End of trial

16

0

week

baseline

**Projektmedicin og procedurer for randomisering og dobbelt-blinding**

Projektmedicinen og placebotabletterne leveres af D3 Pharmacy. Placebokapslerne har identisk udseende som kaps. Cholecalciferol og indeholder de samme fyldstoffer som cholecalciferol kapslerne fraset vitamin D.

Hver deltager vil få udleveret 4 glas med 100 tabletter i hver. En mængde som svarer til 4-5 måneders forbrug.

Center for Clinical and Basic Research pakker og etiketterer medicinen udfra en computergenereret randomiseringsliste. Glassene nummereres fortløbende og udleveres til deltagerne fortløbende som de inkluderes i studiet. Randomiseringslisten opbevares af Hans Christain Høck klinikchef ved Center for Clinical and Basic Research. Ved alvorlige bivirkninger eller hændelser kan randomiseringskoden brydes ved at investigator kontakter Hans Christain Høck og får oplyst deltagerens behandling. I så fald noteres dette i CRF, herunder årsagen til at randomiseringskoden for den pågældende patient er blevet brudt.

**Bivirkninger til behandling med projektmedicin**

Almindeligvis er tilskud af cholecalciferol i de doser som anvendes i nærværende undersøgelse uden bivirkninger. I et studium hvor osteoporose patienter blev behandlet med 18000 IE/dag i fem år, medførte dette ikke en stigning i serum calcium . Risikoen for udfældning af kalksten i nyrerne (nefrokalcinose) anses for at være minimal og patienter med påvirket nyrefunktion kan ikke deltage i studiet

**Compliance**

Under studiets forløb kontrolleres compliance ved tablettælling.

**Medicinsk behandling iøvrigt**

Der må ikke indtages nogen former for kosttilskud under deltagelse i undersøgelsen.

**Metoder**

laboratorieanalyser

forskningsbiobank etableres med henblik på samlet analyse af blodprøver for de parametre der kan tåle denne opbevaring. Øvrige analyseres umiddelbart. Blodprøverne tages fastende ved besøget i uge 0 og uge 16.

*Forsøgsbetingelser*

Den enkelte persons kostindtag skal være den samme i selve urinopsamlingsperioden og 3 dage i forvejen. Dette dokumenteres med kostdagbog i disse dage.

Blodtryksmåling

BT måles med kviksølvsapparat efter Dansk Hypertensionsselskabs retningslinier 2006.

Døgnblodtryksmåling

Smertefri undersøgelse hvor patienten udstyres med særligt blodtryksapparatur, der registrerer blodtrykket løbende gennem 1 døgn. Hvert 15. minut om dagen og hvert 30. minut om natten.

Pulsbølgeanalyse

Et blyantsformet applanations tonometer som indeholder et high-fidelity mikromanometer kan registrere det intraarterielle pulstryk når spidsen appliceres over perifere kar (a radialis, carotis, femoralis, dorsalis pedis). Herved registreres en akkurat trykbølge.

*Pulse wave analysis/Pulsbølgeanalyse:*

På baggrund af 10 sekunders optagelse af pulsbølgen over a. radialis, samt et brachialt blodtryk estimeres pulsbølgen svarende til ascenderende aorta ved hjælp af et valideret software program (SphygmoCor software). Augmentation index (AIx) er et mål for forstærkningen af pulsbølgen med baggrund i refleksionen. AIx beregnes som differencen mellem første og anden systoliske aortikale peak, som procentdel af pulstrykket.

#### *Pulse wave velocity/ Pulsbølgehastighed (PWV):*

PWV bestemmes ved registrering af trykkurver over hhv. a. carotis og a. femoralis til bestemmelse af aortic PWV.

Pulsbølgens transittid bestemmes som tiden mellem R takken i et simultant optaget ekg og ankomsten af trykbølgens intersektive tangent perifert.

Distancen måles som en lige linie mellem registreringsstedet og incisura suprasternalis.

PWV bestemmes ved at dividere distancen med transittiden.

*Forsøgsbetingelser:*

Målingerne foretages efter 10 minutters hvile i rolige omgivelser ved konstant stuetemperatur. Patienten må ikke tale eller sove under undersøgelsen. Målingerne foretages liggende.

Patienten må ikke indtage mad, drikke eller ryge tobak 3 timer forud for undersøgelsen. Alkohol må ikke indtages 10 timer før undersøgelsen.

**Biobank**

For at nedsætte variation mellem analyserne etableres en forskningsbiobank. Ved start besøg samt efter 4 måneder nedfryses 15 ml blod til senere analyse. Blodprøverne tages med standard steril teknik, og mængden svarer til 3 % af hvad der tappes ved en bloddonation. Der er en lille risiko for hæmatom dannelse og infektion i forbindelse med blodprøvetagning. Ved projektets afslutning destrueres materialet.

**Undersøgelsesprogram**

| besøgsuge | forundersøgelse | start / uge 0 | uge 4 | uge 8 | uge 12 | slut / uge 16 |
| --- | --- | --- | --- | --- | --- | --- |
| inklusion/eksklusionskriterier |  | X |  |  |  |  |
| patientsamtale | X | X | X ^1^ | X ^1^ | X ^1^ | X |
| kostregistrering |  | X |  |  |  | X |
| døgnurin: |  |  |  |  |  |  |
| calcium |  | X |  |  |  | X |
| phosphat |  | X |  |  |  | X |
| protein |  | X |  |  |  | X |
| creatinin |  | X |  |  |  | X |
| urinstix: |  | X |  |  |  | X |
| pH |  |  |  |  |  |  |
| blod |  | X |  |  |  | X |
| biokemi: |  |  |  |  |  |  |
| iPTH |  | X^2^ |  |  |  | X^2^ |
| ioniseret calcium | X | X | X | X | X | X |
| phosphat |  | X | X | X | X | X |
| magnesium |  | X |  |  |  | X |
| 25-hydroxyvitamin | X | X^2^ |  |  |  | X^2^ |
| 1,25 dihydroxyvitamin |  | X^2^ |  |  |  | X^2^ |
| triglycerid |  | X |  |  |  | X |
| cholesterol total/HDL/LDL |  | X |  |  |  | X |
| FGF-23 |  | X^2^ |  |  |  | X^2^ |
| creatinin | X |  |  |  |  | X |
| amylase | X |  |  |  |  |  |
| hcg (hos fertile kvinder) | X |  |  |  |  |  |
| basisk fosfatase |  | X |  |  |  | X |
| knoglespecifik basisk fosfatase |  | X^2^ |  |  |  | X^2^ |
| døgnblodtryk |  | X |  |  |  | X |
| blodtryk |  | X |  |  |  | X |
| pulsbølgeanalyse |  | X |  |  |  | X |
|  |  |  |  |  |  |  |
| ^1^ evt telefonisk samtale ^2^ Fryses i forskningsbiobank | |  |  |  |  |  |

**Statistik**

Hovedresultaterne af interventionen vil blive vurderet ved uparret variansanalyse, idet ændringer i den aktivt behandlede gruppe sammenlignes med ændringer i placebo-gruppen. Dette vil foregå dels for den samlede forsøgspopulation, dels i de enkelte strata med justering for kendte confounders.

Derudover vil der blive udført deskriptiv statistik på den samlede patientpopulation, herunder relevante regressionsanalyser.

**Tidsplan**

Rekruttering påbegyndes i uge 36 2009 og fortsætter indtil 40 deltagere er inkluderet. Forsøget forventes afsluttet maj 2010.

**Sted**

Undersøgelserne finder sted i medicinsk ambulatorium, Roskilde Sygehus samt klinisk biokemisk afdeling Roskilde Sygehus.

**Rettigheder/Publikationer**

Samtlige resultater vil blive forsøgt publiceret i internationale peer-review tidsskrifter.

Forfatter rækkefølgen vil være DH, NF, KR, HCH, LB.

Alle resultater, både positive og negative, vil blive offentliggjort.

**Etik**

Oplysningerne om forsøgspersonen beskyttes efter [lov om behandling af personoplysninger](https://www.retsinformation.dk/Forms/R0710.aspx?id=828) og [sundhedsloven](https://www.retsinformation.dk/Forms/R0710.aspx?id=114054).

Denne undersøgelse frembringer information om et kosttilskud som allerede kan købes og anvendes af hele den danske befolkning. D vitamin i denne høje dosis kan muligvis medføre forandringer i mineralmetabolismen. Denne undersøgelse skal afklare om dette er tilfældet. Da forsøgsdeltagerne har et lavt D vitamin niveau forventer vi ingen overbehandling. Vi vil alligevel være opmærksomme på en eventuel stigning i calcium. I den forbindelse informeres deltagerne om at symptomerne på høj calcium er tørst og større og hyppigere vandladninger. På længere sigt kan høj calcium medføre nyresten, knogleafkalkning, psykisk ubalance og forstoppelse men dette forventes ikke da vi følger plasmacalcium med blodprøvekontrol hver måned. Høj calcium har dog ikke tidligere været beskrevet at udvikle sig hos raske personer behandlet med denne dosis D vitamin.

Den enkelte forsøgsdeltager får ved deltagelse en viden omkring sit plasma D vitamin niveau og ved D vitamin mangel korrigeres dette under tæt kontrol. Vi opnår væsentlig fysiologisk og farmakologisk information om hvordan mineralmetabolismen forandres når plasma D vitamin niveauet øges til det af mange anbefalede højere normalområde.

Deltagerne er raske frivillige forsøgspersoner som ikke nødvendigvis havde modtaget D vitamin behandling hvis de ikke indgik i studiet da D vitamin betragtes som et kosttilskud og deltagerne i øvrigt er raske forsøgspersoner. Deltagerne vil således ikke være dårligere behandlet fordi de modtager placebo i undersøgelsens 4 måneder.

Da vi ikke kender risikoen for forhøjet udskillelse af calcium og fosfat i urinen ved højdosis D_3_ vitamin behandling, vil vi med vores undersøgelse vise om der indenfor de første 3 måneder sker en målelig ændring.

I tilfælde af at vi finder en målelig ændring som vi på længere sigt finder betænkelig med risiko for udvikling af bl.a. nyresten må vi anbefalede reduceret dosis af D3 vitamin.

Ved afsluttet deltagelse vil deltagerne blive individuelt vejledt med hensyn til fremtidig behov for D vitamin tilskud.

## Deltagerinformation

Deltagerinformation og rekrutteringsopslag indeholder telefonnummer på kontaktperson hvorfra der kan rekvireres yderligere information under hele forsøget.

Når interesserede deltagere henvender sig med baggrund i opslag informeres de kort telefonisk om projektet, samt om at der er tale om et medicinsk forsøg, af lægerne Niels Erik Frandsen eller Ditte Hansen. Deltageren tilsendes en skriftlig deltagerinformation. Der aftales et tidspunkt for mundtlig information hvor deltageren kan medbringe en bisidder. Informations mødet foregår i rolige uforstyrrede rammer i medicinsk ambulatorium. Mødet planlægges således at deltageren har minimum 5 dage til at læse deltagerinformationen.

Deltageren informeres i en forståelig fremstilling om forsøget og dets forudsigelige risici, bivirkninger, komplikationer, samt at der kan være uforudsigelige risici og belastninger knyttet til at deltage i et forskningsprojekt. Informerende læge har ansvaret for at informationen er forstået.

Herefter bedes deltageren snarest muligt dog efter minimum 1 døgns betænkningstid tage stilling til sin deltagelse. Ved ønske om deltagelse kontaktes de forsøgsansvarlige på ny og der aftales tid for 1. besøg hvor underskrevet samtykkeerklæring medbringes

Deltager kan ved underskrift på samtykket fravælge viden om væsentlige helbredsoplysninger fremkommet ved undersøgelsen.

Forsøgspersonen skal informeres såfremt der under projektets udførelse kommer nye oplysninger om effekt, risiko, ulemper, bivirkninger, komplikationer, eller hvis der ændres væsentlig i forsøgsdesign, og der skal indhentes fornyet samtykke. Desuden informeres patienten hvis der i forbindelse med forsøget fremkommer væsentlige oplysninger om den enkelte persons helbredstilstand, medmindre denne udtrykkeligt giver udtryk for ikke at ønske denne information.

Hvis ny viden om bivirkninger og risici medfører, at proceduren ved forsøget straks ændres, udarbejdes en revideret skriftlig patientinformation, som forelægges den videnskabsetiske komité til godkendelse. Efterfølgende informeres forsøgspersonerne, og der indhentes på baggrund af informationen et nyt skriftligt samtykke til deltagelse.

Ved forsøgets afslutning informeres forsøgspersonerne om resultaterne, samt eventuelt konsekvens for den enkelte. Dette sker så vidt muligt per brev.

**Dataindsamling**

Data indtastes direkte i database. Databasen anmeldes til datatilsynet via regional fælles anmeldelse.

**Finansiering**

Studiemedicinen leveres gratis af D3 Pharmacy Denmark.

25 hydroxyvitamin D analyserne udføres og sponsoreres af Nordic Bioscience, Herlev Hovedgade 207, 2700 Herlev..
Øvrige analyseudgifter betales af nefrologisk afdeling Roskilde samt om muligt ved støtte fra private og offentlige fonde.

Pakning og etikettering foretages gratis af Center for Clinical and Basic Research efter aftale med Hans Christian Høck klinikchef.

De forsøgsansansvarlige har ingen økonomisk tilknytning til D3 Pharmacy Denmark.

Der udbetales ikke vederlag til forsøgspersoner.

Reference List

1. Moller UK, Ramlau-Hansen CH, Rejnmark L, Heickendorff L, Henriksen TB, Mosekilde L: **Postpartum vitamin D insufficiency and secondary hyperparathyroidism in healthy Danish women.** *Eur J Clin Nutr* 2006, **60:** 1214-1221.

2. Mosekilde L: **Vitamin D and the elderly.** *Clin Endocrinol (Oxf)* 2005, **62:** 265-281.

3. Mosekilde L, Brot C, Hyldstrup L, Mortensen LS, Molgard C, Rasmussen SE *et al*.: **[The vitamin D status of the Danish population needs to be improved].** *Ugeskr Laeger* 2005, **167:** 895-897.

4. Bischoff-Ferrari HA, Giovannucci E, Willett WC, Dietrich T, wson-Hughes B: **Estimation of optimal serum concentrations of 25-hydroxyvitamin D for multiple health outcomes.** *Am J Clin Nutr* 2006, **84:** 18-28.

5. Hoeck HC, Li B, Qvist P: **Changes in 25-Hydroxyvitamin D3 to oral treatment with vitamin D3 in postmenopausal females with osteoporosis.** *Osteoporos Int* 2008.

6. Armas LA, Hollis BW, Heaney RP: **Vitamin D2 is much less effective than vitamin D3 in humans.** *J Clin Endocrinol Metab* 2004, **89:** 5387-5391.

7. Hasling C, Nielsen HE, Melsen F, Mosekilde L: **Safety of osteoporosis treatment with sodium fluoride, calcium phosphate and vitamin D.** *Miner Electrolyte Metab* 1987, **13:** 96-103.

8. Forman JP, Giovannucci E, Holmes MD, Bischoff-Ferrari HA, Tworoger SS, Willett WC *et al*.: **Plasma 25-hydroxyvitamin D levels and risk of incident hypertension.** *Hypertension* 2007, **49:** 1063-1069.

9. London GM, Guerin AP, Verbeke FH, Pannier B, Boutouyrie P, Marchais SJ *et al*.: **Mineral metabolism and arterial functions in end-stage renal disease: potential role of 25-hydroxyvitamin D deficiency.** *J Am Soc Nephrol* 2007, **18:** 613-620.

10. Lind L, Wengle B, Wide L, Ljunghall S: **Reduction of blood pressure during long-term treatment with active vitamin D (alphacalcidol) is dependent on plasma renin activity and calcium status. A double-blind, placebo-controlled study.** *Am J Hypertens* 1989, **2:** 20-25.

11. Pfeifer M, Begerow B, Minne HW, Nachtigall D, Hansen C: **Effects of a short-term vitamin D(3) and calcium supplementation on blood pressure and parathyroid hormone levels in elderly women.** *J Clin Endocrinol Metab* 2001, **86:** 1633-1637.

12. Escribano J, Balaguer A, Pagone F, Feliu A, Roque IF: **Pharmacological interventions for preventing complications in idiopathic hypercalciuria.** *Cochrane Database Syst Rev* 2009, CD004754.

13. Altman D.G.: *Practical Statistics for Medical Research*. 1991.
